# A study of socio clinical, biochemical and electrocardiographic changes of yellow oleander seed poisoning in India

**DOI:** 10.6026/97320630019

**Published:** 2023-03-31

**Authors:** Murugan Subramani, Mudali Anbarasan, Shanmugam Deepalatha, Lakshmi Narayanan Muthumani, Mallu Azmeera Mahesh

**Affiliations:** 1 Department of General Medicine, K.A.P Viswanatham Govt Medical College, Trichy - 17, India; 2 Department of Microbiology, Thanjavur Medical College, Thanjavur, Tamilnadhu, India

**Keywords:** *Thevetia peruviana*, Toxicity, cardiotoxicity

## Abstract

Yellow oleander (*Thevetia peruviana*), which belongs to the Apocyanaceae family, is a common shrub seen throughout the tropics. All parts of the plant contain high concentrations of cardiac glycosides, which are toxic to cardiac muscle and the autonomic nervous system. The main objective of this study was to socio-clinical, biochemical and electrocardiographic changes of yellow oleander seed poisoning. This prospective observational study was conducted over a period of 6 months (March 2022 to September 2022). Oleander seed poison in most prevalent in the 21-40 years age. More the crushed seeds consumed and the delay to admission to the hospital for treatment poorer was the outcome. The most common GI symptoms of yellow oleander poisoning were vomiting (58 %), abdominal pain (28%), diarrhoea (9%), and palpitations (20%), dizziness (18%).Serum potassium levels that were measured during the admission were directly related to the ECG changes. ECG changes were more observed with patients those who consumed seeds in crushed form and this difference is statistically significant (P = 0.0001). Higher incidence of cardiotoxicity was noted with patients those who consumed poison on empty stomach compare to who consumed after food. The Electrocardiographic manifestations was found even with consumption of one seed, number of seeds consumptions independent of cardiotoxicity.Additonally higher mean Potassium value observed in patients who had cardiotoxicity when compared to patient who had no cardiotoxicity. Death of the patients in yellow oleander seed poisoning was independent of quantity of the seeds they have ingested.

## Background:

Yellow oleander (*Thevetia peruviana*), which belongs to the Apocyanaceae family, is a common shrub seen throughout the tropics. Deliberate self-harm by consumption of this plant is a common toxicological emergency in South Asian countries, especially in India and Sri Lanka [1,2,3]. Ingestion of any part of this plant is toxic, as all parts contains a variety of cardiac glycosides including neriifolin, thevetin A, thevetin B, oleandrin, and other unidentified substances [4]. Ingestion of yellow oleander (*Thevetia peruviana*) results in clinical symptoms similar to those of digitalis toxicity [5]. Common symptoms include nausea, vomiting, abdominal pain, diarrhea, dysrhythmias, and restlessness. A common electrolyte abnormality is hyperkalemia. Cardiac toxicity, due to the cardiac glycosides, is a common life-threatening clinical manifestation. Patients may develop bradycardia with atrioventricular (AV) block, atrial tachycardias, ventricular tachycardia including bidirectional ventricular tachycardia, and ventricular fibrillation. Cardiogenic shock with myocardial depression can also occur. The arrhythmogenic effects of the cardiac glycosides are caused due to a combination of the direct effects of the toxin on the myocardium and the conducting system of the heart and the neurally mediated increases in autonomic activity [6]. Ingestion of seeds causes severe problem (up to lethal) due to higher content of glycosides in thevetin compared to the other parts of the plant. Roberts et al. [7] reported that the percentage of glycosides existed in the seeds is 4.800 followed by leaf, latex and fruit of yellow oleander as 0.070, 0.045 and 0.036 respectively. The cardiac glycosides inhibit the transmembrane Na+ /K+ ATPase pump, and this action produces increased intercellular concentrations of Ca++ and Na+. Sub endocardial and perivascular haemorrhage along with focal myocardial edema are the common pathological findings during examination of fatal cases of yellow oleander poisoning [8]. *Thevetia peruviana* is a tropical, evergreen, small ornamental tree/shrub grown in gardens and roadsides, which grows to about 1.5 - 2.3 m height. The leaves are spirally arranged, linear and about 13- 15 cm in length [8]. Flowers are bright yellow and funnel-shaped, sometimes fragrant, with five petals spirally twisted. The fruits are somewhat globular, slightly fleshy and have a diameter of 4- 5 cm. The fruits, which are green in colour, become black on ripening .Each fruit contains a nut, which is longitudinally and transversely divided. Cardiac glycosides exert various direct cardio toxic effects through a variety of mediators such as histamine, nitric oxide, leukotrienes, and endothelin, angiotensin, and superoxide radicals. Increased central sympathomimetic activity on the heart also plays an important role in the development of cardiac arrhythmias in patients with cardiac glycoside poisoning. Hence, the use of parasympathetic system blockade with atropine, or the use of β-adrenergic agonists, may result in tachyarrhythmia [9]. Severe hyperkalaemia can contribute to atrioventricular (AV) block and depressed myocardial excitability. Dysrhythmias often associated with cardiac glycoside toxicity include Brady dysrhythmias, sinus bradycardia with all types of AV nodal block, junctional rhythms, and sinus arrest. Dysrhythmias characterized by increased automaticity and conduction blockade are highly suggestive of cardiac glycoside toxicity. These include tach dysrhythmias such as atrial tachycardia with block, paroxysmal atrial tachycardia with block, bidirectional ventricular tachycardia, and ventricular fibrillation [10]. The objectives of this study were to identify the various cardiac arrhythmias and electrolyte abnormalities in yellow oleander poisoning. This study was also designed to identify clinical and biochemical parameters at presentation which can predict serious arrhythmias. In most cases, clinical management of poisoning, by yellow oleander (T. peruviana), involves administration of activated charcoal and supportive care including temporary pacemaker insertion. This study aimed to describe the clinical profile and outcomes among patients with yellow oleander poisoning requiring admission to a tertiary care centre in South India [11]. The objective of this study were to study the Demographic and Biochemical profile and the ECG changes associated in Yellow Oleander seed poisoning .This study also correlates the number of seeds and the form of consumption of seeds with the severity of the ECG changes identified.

## Materials and Methods:

## Study settings and population:

This observational study was conducted with sample size of 100 patients admitted in the Department of General Medicine, Mahatma Gandhi Memorial Hospital, Trichy from March 2022 to September 2022 Patients more than 18 years of age ,admitted with the consumption of yellow oleander seed poisoning within 48 hours.

## Methods:

All patients admitted with consumption of yellow oleander seed poison within 48 hours in the Department of General Medicine, MGMGH, Tiruchirappalli were studied. Selected socio-demographic, detailed Clinical, Biochemical and Electrocardiographic changes were recorded in addition to detailed history of number of seeds consumed, time of consumption, time of presentation to hospital; treatment details prior to admission were done. Patients were monitored for Electrolyte imbalance, continuous cardiac monitoring for recognizing Cardiac Arrhythmias and Outcome of the Patient condition were recorded. Selected sociodemographic, clinical, biochemical, electrocardiographic, and treatment details were collected from the patients and recorded in a questionnaire. Data regarding poisoning comprised of part ingested, quantity of poison, method of ingestion, whether consumption in empty stomach or after food, the intention behind poisoning, time of ingestion, first aid at home, consumption to admission interval, treatment given, duration of hospital stay, and the type of outcome. 12-lead ECG including rhythm strip was taken at admission before instituting treatment and repeated depending on the clinical status. Ethical committee approval obtained from the institutional ethical committee. An informed consent was obtained from all patients who were included in the study.

## Inclusion criteria:

[1] Those who consumed Yellow Oleander seed.

[2] Age more than 18 years.

## Exclusion criteria:

[1] Age less than 18 years of age.

[2] Those with underlying severe cardiac, renal or hepatic disease.

[3] Patients who had ingested plant parts other than the seed.

[4] Patients who were known cases of Dyselectrolytemia.

[5] Patients who were taking following drugs:

1) Digoxin, diuretics, verapamil, beta-blockers,ACE inhibitors, Amiodarone, calcium and potassium supplements.

2) Brought dead patient and those died before indoor treatment.

## Statistical analysis:

Data were entered in Microsoft Excel spreadsheet and analysed utilizing the software - Epidemiological Information Package 2002 - developed by the Centre for Disease Control and Prevention, Atlanta, for the World Health Organization. Frequencies, percentages, range, mean, standard deviation, and "P" values were calculated using this package. Chi-square test was performed to find out the significance of the relationship between the groups The difference was considered to be statistically significant if "P" < 0.05.

## Results:

Upon analysing, the data collected from the 100 sample of patients who were included in the study after consuming the oleander seed poisoning a total number of admissions in General, Medicine Mahatma Gandhi Memorial Hospital, Trichy, during the study period, was 11.1 per 1000 admissions. It fairly accounted for 91.7% of the cases admitted for plant poisoning. 10% of the cases among the total number of admission in general medicine wards were poisoning cases. Among the poisoning cases, 87.2% were chemical poisoning and 12.8% plant poisoning where yellow oleander accounted for 10.1% of the cases. The maximum number of cases occurred in the age group of 21-40 years ( 57 % ) followed by the age group of 41-50 years ( 22%) and greater than 51 years of age (11 %), respectively. The age of the patients ranged from 18 to 63 years. The mean age and standard deviation was 36.6 ±10.6. Male to Female ratio 1:1.3, but this gender difference was not significant statistically among the 100 cases, 43 were males and 57 were females (Table 1). In this study conducted among 100 cases 41 % cases belongs Upper lower socioeconomic class, followed by 30% cases belongs to Lower socioeconomic class, only 6 % cases belongs to Upper middle class. The intention behind poisoning was suicidal in majority of cases (81%) and accidental in 7% of the patient population. No cases of homicidal poisoning were reported. All of the patients had consumed the seeds of the yellow oleander plant. The majority of the patient population (70%) have taken less than five seeds and (30 %) have taken more than five seeds [Table 2]. from the 100 cases, number of seeds consumed range between 1-10. Maximum number of 10 seeds was consumed by two cases. Mean number of yellow oleander seeds consumed was 4.43 ±2.12, minimum number of one seed consumed by 8% of patients, majority of patients consumed four seeds (21%). The method of ingestion of the seeds were crushed in 52 % of the cases, chewed in 29 % and swallowed in 19% were 54 % of the patients have consumed the poison in the empty stomach and 46 % have consumed after food intake .The difference in the group was statistically significant (p=0.0001). The most common GI symptoms of yellow oleander poisoning were vomiting (58 %), abdominal pain (28%), diarrhoea (9%), and palpitations (20%), dizziness (18%).Among 100 patients, other symptoms were cardiovascular symptom like palpitations (20%) dyspnoea (5%), and few patients had dizziness (18%). Only one patient presented with altered mental status.58% patients after consumption of yellow oleander seeds presented to hospital with complaints of vomiting. Vomiting in yellow oleander poisoning has significant value (p<0.05) in relation to Electrocardiographic changes. Serum potassium levels were measured at the time of admission and there mean value was 4.54 ±0.86.Of these 100 cases only 3 cases had hyperkalaemia, 8 cases had hypokalaemia and in majority (89.2%) serum potassium values were within normal limits. The serum Potassium values ranges from 3.3 to 6.1 and mean value of serum Potassium slightly was higher. There is significant correlation between electrocardiographic changes and serum Potassium (Table 3). On studying the correlation between the age group and the ECG changes Out of 10 patients of less than 20 years age, 5 patients developed ECG changes. Majority of the patients had ECG changes belongs to age group 21-40. 9 patients ECG changes out of 22 in 41-50 years age group were observed. Out of 11 patients in age group more than 51, 7 patients had ECG changes. In our study, ECG changes in relation to age group was statistical significant (p=0.044). In our study out of 100 patients, 35 patients were admitted within 6 hours of seed consumption, in those patients, 42.80% had abnormal ECG changes, 51.20% of patients got admitted in 6-12 hours after poison consumption is 45, out of which 73.3% patients developed ECG changes, 26.7% patients had normal ECG. Number of patients presented 12 hours after the poison consumption is 20, out of this 75%, patients had ECG changes and 25 % had normal ECG findings. In our study correlation between time interval for admission to ECG changes is significant statistically (p=0.009) [Table 4]. In our study among 100 patients, 52 patients consumed oleander seeds in crushed form. Out of this 80.70% were noted. Patients developed ECG changes, 29 patients consumed in chewed form in which 51.70% patients developed ECG changes, 19 patients consumed in swallowed form in this group 31.40% had ECG changes. ECG changes were more observed with patients those who consumed seeds in crushed form and this difference is statistically significant (P = 0.0001) (Table 4).

## Treatment and Outcome:

In our study out 100 patients, gastric lavage was given in 90 % of the cases .In those who were given gastric lavage ,39% did not develop any cardiotoxicity compared to 33.3% in those who were not given a gastric lavage .The difference between the groups was statistically insignificant (P=0.5675). 81% patients were improved and discharged well. 9% patients absconded from medical wards and 7% patients were against medical advice. Among 100 patients death occurred in 3% patients. The mean duration of the hospital stay was 4.65 days ranging from 1-10 days .The mean duration in no, some and severe cardiotoxicity groups were 3.64,4.96 and 5.65 days.The difference between the groups was statistically significant (p=0.0001).

## Discussion:

Yellow oleander poisoning is a common form of plant poisoning in South Asia. All parts of the plant are poisonous, especially the seeds and leaves. Deliberate self-harm by consuming yellow oleander seeds is common in young women. The number of seeds consumed in our study was not associated with increased mortality, which is similar to the findings of Bose et al. [7]. In our study, gastrointestinal toxicity was a common presenting symptom including vomiting and nausea. Among the 100 cases study population, the mean age of patients was around 36.6± 10 years. Studied population age range from 18-63 years. Majority number of cases belongs to age group from 21-40 years. Few numbers of cases belongs to age above 60 years. Our study observations coincide with the previous Srilankan and Indian studies. In those studies, also reported that Yellow Oleander poisoning common among young adults[2], Srilankan study, in this study, 415 cases observed and most of the patients were young and mean age was 32.4 years, age of the study population range from 11-71 years [2]. Study also reported that more than 50% of patients were Females. Regarding sex distribution in this study, 53.8% of the patients were Females and Males were 46.2%. Female to Male ratio is 1.16:1. In our study, Gender distribution almost similar [2]. In our study population out 100 cases, 57% were Females and 43% cases were Males, Female to Male ratio in our study population is 1.3:1. Few other Indian and Srilankan studies also reported that there is a slight Females preponderance when compared with Males. In our study population majority of the patients were from rural areas and fact that oleander plant widely grown in rural areas and it is easily available for rural population. there was no need of spend money for consuming this poison hence this is one of the most common form of poisoning in rural areas and for many patients this was the only toxic plant known to them . According to modified kuppusamy socioeconomic scale majority of the patients in our study population upper lower class and lower class, majority of them were wage daily labourers and very few patients belongs to upper middle class. In our study population majority of the cases around 81%, the intention behind the consumption of poison is suicide. The reasons included for suicide were unemployment, depression, academic pressure, alcohol consumption, failure to get success in their respective fields, harassment, inter personal conflict, in teenagers and adoloscents mainly due to love failure, unable to accept loss of beloved ones. There was 7% of cases consumed poison accidentally. This accidental poisoning observed among adolescent age group as well as elderly age group, they were consumed poison due to mis-interpretation of the substance. In 12% of the cases attempt of the poisoning was done just to frighten or blackmail others for some personal benefit or to resolve conflicts among them. There is no homocidal poisoning reported in our study. In our study observations regarding reason behind poisoning similar to that reported by study conduct at coimbatore medical college hospital. In our present study included patients those who consumed seed only, in which majority 52% of the patients consumed seeds in crushed form, 80.70% of these patients had electrocardiographic changes. In chewed out 29 patients 51.70% patients developed ECG changes, in swallowed form of consumption out of 19 patients 31.40% patients had ECG changes. Higher incidence of electrocardiographic changes observed with patients those who consumed seeds in crushed form compared to other form of consumption. This difference may be due to high amount of cardiac glycosides available for absorption when seeds consumed in crushed form. This observation was supported by Srilanka people generally eat seeds as whole and develop lesser extent of cardiotoxicity when compare to crushed and chewed mode of consumption. Similar observations reported in [3] study. In this study patients those who consumed seed in empty stomach higher in number compare to those who consumed poison in association with food, either with mixing food or after food intake, but incidence of electrocardiographic changes seen more with patients those taken seeds on empty stomach. This is most probably due to the absorption cardio glycosides better in empty stomach [3] study similar results were assessed.

The symptoms of yellow oleander seed poisoning in our study population were vomiting 58%, abdominal pain 28%, diarrhoea 9%, and palpitations 20%, dizziness in 18 % patients were observed, only one patient presented to our emergency department with altered mental status. This concordance results [5] they reported that vomiting, diarrhea and dizziness were the most common presenting symptoms of their study of 170 Srilankan cases. In our study, more cardiotoxicity seen with patients those had vomiting, abdominal, and palpitations compared to those do not have these symptoms. In our study, vomiting and abdominal pain had significant correlation with cardiotoxicity (reported similar correlation results in his study among 106 cases of [8]. Gastrointestinal symptoms mainly vomiting, abdominal pain more commonly seen with the patients those who consumed seeds in crushed form, compare to chewed, and swallowed form of consumption. This is probably due to in crushed form cardiac glycosides can easily cause the gastric irritation and stimulates the chemo trigger zone. In our study population, this correlation was statistically significant. Similar findings also reported in [9].

Seeds of the yellow oleander were more toxic than other parts of plant. In oleander seed, poisoning one of the most debated area is the toxic dose of the seeds. In our study population number of the seeds ingested range from one seed to 10 seeds. Among 100 cases single seeds consumed by 8 patient, two seeds consumed by 13 patients, more number of patients consumed seeds in range from 2 to 6, only one patient consumed 9 number of seeds and 10 number of seeds consumed by two patients. Mean number of seeds consumed in our study population is 4.42 with standard deviation of 2.02. Among 100 cases number of patients consumed seed upto 5 is 70 out of which 60% patients developed cardiotoxicity and 40% without cardiotoxicity, remaining 30 patients consumed more than 6 numbers of seeds in this group 70% of patients developed cardiotoxicity, 30% of the patients without cardiotoxicity. Incidence of electrocardiographic changes slightly higher in patients who consumed more number of seeds but patients who ingested only one seeds also had ECG changes similar to the patient who ingested more number of seeds like 7, 8,9,10. This observations were concordance with (8) among 415 Srilanka cases and (9) among 51 patients. These studies also reported no significant relationship with seeds and electrocardiographic changes. In our present, study even no significant relationship between number of seeds and outcome. Three patients died in our study population due to severe poisoning. Three patients who died had ingested seeds 3, 4, 5 respectively. Those studies also reported that there is no significant relationship between number of seeds consumed and outcome. In (3) study six patients death occurred and those patients who died had consumed seeds 10, 5, 8, 1, 5, 2 respectively [8]. Study two patients died and they consumed seeds 5 and 18 respectively. The quantity of oleander poison cannot determine outcome and toxicity [10]. It may be associated with some other factors like time interval between consumption and admission, for of consumption, ingestion on empty stomach or not, delay in first aid and treatment etc.

Time interval between poison consumption to hospital admission was the one of the important determining factor of outcome and cardiotoxicity in yellow oleander seed poisoning. In our study population time interval between poisoning and admission was categorized as those who presented to hospital less than 6 hours of consumption and those who presented in between 6 to 12 hours, those presented after 12 hours of poison consumption. 35 cases presented to hospital within 6 hours of which 52.20% had normal ECG, 45 cases presented to hospital in-between 6- 12 hours of which 73.3% had abnormal ECG, maximum 75% ECG abnormalities recorded in patients those were presented 12 hours after consumption of poison. A positive correlation obtained in our study between delay in getting admitted to abnormal ECG changes. The reason for delay in admission was lack of adequate transportation facility in interior rural areas. In high intention, suicide patients relatives come to about poisoning only several hours after the consumption of poison, these factors may contribute to delay in getting admission to our hospital [10]. In our study population the electrocardiographic abnormalities were noted mainly because of increased refractiness of atrioventricular pathway and also depressed atrioventricular conduction. Among 100 patients 37%, patients had normal ECG, and 63% patients were developed electrocardiographic abnormalities. In our study, most common electrocardiographic abnormalities were sinus bradycardia, T wave inversion, ST segment depression, first-degree atrioventricular block, third degree of consumption, ingestion on empty stomach or not, delay in first aid and treatment [11]. Time interval between poison consumption to hospital admission was the one of the important determining factor of outcome and cardiotoxicity in yellow oleander seed poisoning. In our study population time interval between poisoning and admission was categorized as those who presented to hospital less than 6 hours of consumption and those who presented in between 6 to 12 hours, those presented after 12 hours of poison consumption. 35 cases presented to hospital within 6 hours of which 52.20% had normal ECG, 45 cases presented to hospital in-between 6- 12 hours of which 73.3% had abnormal ECG, maximum 75% ECG abnormalities recorded in patients those were presented 12 hours after consumption of poison. A positive correlation obtained in our study between delays in being admitted to abnormal ECG changes. The reason for delay in admission was lack of adequate transportation facility in interior rural areas. In high intention, suicide patients relatives come to about poisoning only several hours after the consumption of poison, these factors may contribute to delay in getting admission to our hospital [12]. In our study population, the electrocardiographic abnormalities were noted mainly because of increased refractiness of atrioventricular pathway and depressed atrioventricular conduction. Among 100 patients 37% patients had normal ECG, and 63% patients were developed electrocardiographic abnormalities. In our study, most common electrocardiographic abnormalities were sinus bradycardia, T wave inversion, ST segment depression, first-degree atrioventricular block, third degree atrioventricular block or complete heart block, tall T wave, Sino atrial block. The findings were concordance to that of (4) also reported tachyarrhythmia's like atrial fibrillation, atrial flutter, ventricular fibrillation, ventricular tachycardia these ECG abnormalities not observed in our study, [12] also described regarding 3-6 % of supraventricular tachycardia and ventricular ectopic, these observations also not noted in our study group. Dysrhythmias occurred in yellow oleander seed poisoning in which bradyarrhthmias more common compare to tachyarrhythmias due to depressed atrioventricular conductions. ECG abnormalities of yellow oleander seed poisoning differs from digoxin toxicity in a way that in digoxin toxicity tachyarrhythmia is more common compared to bradyarrhthmias where as in yellow oleander poisoning bradyarrhthmias more common. Electrolyte abnormalities also occurred in yellow oleander seed poisoning, and this electrolytes abnormalities also one of the determine factor of toxicity and outcome. In our study, population normal serum potassium reference value taken as 3.6 to 5.2 meq/L. The mean value of serum potassium in our study population is 4.54, the range from 3.3 to 6.1, the patients those who were presented with increased serum potassium values were 19. Out of this 89.47% patients developed abnormal ECG changes. [13]. High potassium levels further precipitate the cardiac arrhythmias. There was a significant correlation between serum potassium levels and abnormal ECG changes in our study population. Similar observations noted in most of the Indian and continental studies. (3, 4) study reported that few cases with very severe form of hyperkalemia like 10.8meq/L, 7.2meq/L, 8.8 meq/L. In our study three patients showed low baseline potassium value, similar findings also noted in [14.] in that study 9 patients had hypokalemia. Hypokalemia in oleander poisoning is mostly due to persistent vomiting and this vomiting occurred either due to poisoning per se or due to induced emesis. Hypokalemia can also potentiate the toxicity. Hypokalemia and hyperkalemia are both notorious in yellow oleander-poisoning [14] serial monitoring of potassium necessary for treating oleander seed poisoning. In our 100 study population death occurred in three patients, in which one male patient and two female patients were died. Out of three deaths, two deaths occurred within one hour of admission and another death occurred one day after the admission. In eastern part of the India (10) reported case fatality rate of 4.6% among 300 patients [15]. Also, observed case fatality rate 10%. Lower case fatality noted in our study population probably due to poisoning severity low in Tamilnadu when it compared to Srilanka.

## Limitations of the study:

1) It is a single centred study.

2) Sample size to be increased.

3) Age less than 18 years individuals were not included in our study.

4) In our study, Patients were not tested for serum cardiac glycosides levels.

5) Digoxin specific Fab fragment antibodies not used in our study for treating patients.

## Figures and Tables

**Table 1:** Age and gender wise distribution of study population

**Age group**	**Male (43)**	**Female (57)**	**Total**	**Mean Age±SD**
<20	4	6	10	
21-40	27	30	57	36.6±10.6
41-50	9	13	22	
>51	3	8	11	
Total	43	57	100	

**Table 2:** Manner, Mode and number of seeds consumed and the time interval between consumption and admission and symptoms caused by oleander seed poisoning

S.No	**Variable**	**No of patients**	**Others Symptoms(among the 100 patients)associated with cardiotoxicity**				
			**Vomiting**	**Abdominal pain**	**Diarrhoea**	**Palpitations**	**Dizziness**
1	Empty stomach	54	58%	28%	9%	20%	18%
2	After food intake	46					
3	Crushed	52					
4	Chewed	29					
5	Swallowed	19					
6	No of seeds less than 5	70					
7	No of seeds more than 5	30					
8	Less than 6 hours	35					
9	6-12 hours	45					
10	More than 12 hours	20					

**Table 3:** Descriptive Statistics for the biochemical profile of the patients with yellow oleander seed poisoning

**Biochemical Parameters**	**N**	**Minimum**	**Maximum**	**Mean**	**Std. Deviation**
Blood sugar		89	168	120.1	15.5
Blood Urea		15	40	27.52	5.85
Serum Creatinine		0.7	1.8	1.03	0.22
Serum Sodium		12	5	9	8.72
Serum Potassium	100	3.3	6.1	4.54	0.86

**Table 4:** Correlation between ECG changes and window period, quantity of seeds consumed, manner of consumption and serum potassium

**ECG**	**Normal**		**Abnormal**		**Total**	**p Value**
	**N%**	**N**	**%**		
Window period:					
Less than 6hrs	2051.20%	15	42.80%	35	
6-12 hrs	1226.75%	33	73.30%	45	
More than 12 hrs	525%	15	75%	20	
Total	3737%	63	63%	100	0.009
Number of seeds Consumed :					
1-5 seeds	2840%	42	60%	70	0
More than 6 seeds	930%	21	70%	30	
Total	3737%	63	63%	100%	0.34
Form of consumption :					
Crushed	1019.20%	42	80.70%	52	
Chewed	1448.20%	15	51.70%	29	
Swallowed	1368.40%	6	31.40%	19	0.0001
Total	3737%	63	63%	100	
Serum Potassium:					
Normal Potassium Values	3543.20%	46	56.79%	81	
Abnormal Potassium Values	210.52%	17	89.47%	19	
Total	37	63		100	0.008
